# The Old and the New

**Published:** 1877-01

**Authors:** Henry S. Chase

**Affiliations:** St. Louis, Mo.


### ARTICLE VIII.

### *The Old and the New.*

BY HENRY S. CHASE, M. D., ST. LOUIS, MO.

**Read at the Eighth Annual Union Meeting of the 7th and 8th District Dental Societies, held at Rochester, N. Y., Oct. 31st, and Nov. 1st, 1876.**

It is interesting to observe the change of fashions, opinions and modes of manipulation in dentristy, as well as in other professions besides our own.

About thirty years ago, the methods of practice, in many respects, were what they are *becoming* now. For it must be evident, to most observing minds, that quite a marked change is going on in Operative Dentistry to-day. Our profession seems to be pnogressing in a circle or spiral, and not in a straight line. This is indeed true of nearly all improvements in social life, in thought, custom, and government.

We have progressed in a circle which has carried us to a *higher plane* of dental skill; and we must not think that we have *retrograded* merely because we can now see a portion of the same landscape, that we so well knew thirty years ago. Thirty five years ago *amalgams* were extensively used, and more teeth were filled with tin-foil than with gold.

Non-cohesive gold-foil was used in cylinders and ropes. Sound teeth were filled apart and polished, to prevent future decay. Filing was freely indulged in where proximal cavities were to be plugged, and it was considered important that teeth should be sufficiently separate, after filing, to admit of the ready removal of food from between them.

Teeth were occasionally extracted and reset in the socket, to cure periostitis. The very young practitioner to-day has been taught that a great deal better *work* is done *now* that it was thirty years ago, and that it is owing to an improved mode of manipulation.

Well, that is partly true and partly not true. I am satisfied that teeth containing gold fillings and tin fillings *then* had a *longer life* than those which have been filled and refilled during the last fifteen years of cohesive foil. A large proportion of the former had *water-tight* plugs; which cannot be said of the last decade, I believe.

On the other hand, preparation of cavities is more thorough to-day than in olden times; for *fissures* were not as universally cut out then as now. But our thoroughness of preparation *has* not been taken advantage of, by having water-tight plugs invariably. *Now* we unite the two by the use of *cylinder* fillings. *Now* we discriminate in the use of tin, by placing such plugs in *proximal* cavities, only.

Now we *line* the walls of grinding cavities with *tin-foil*, and use gold for the more central portion; which thereby prevents the *wear* of the tin, lessons the force of the electric current from the dentos to the plug, and insures a water-tight plug in a higher degree than where gold is alone used

*Now*, instead of using a black and disgusting looking amalgam, which shrinks, leaks and discolors the dentos, we employ materials in our alloy which are purer and oxidise less; making it of constant proportions; which does not contract in hardening; does not leak; maintains a good color in nearly all mouths; has an electric current from the dentos as feeble as tin, and which I believe prevents future decay as well as any other metal or combination of metals known.

But when I thus speak of an amalgam I do not include *all*, or indeed any large proportion of the alloys at present in the market. From *experience* I can only include two in this commendation.

*Now*, instead of the tedious use of the file we cut teeth apart with disks of corundum, diamond-copper, rubber, etc.

Besides these modifications of Old Practice, there have been many, very many, *new* and valuable inventions, for which we are grateful and proud.

And so, we have progressed on the plane of a *spiral;* going up higher all the time, and yet coming again into close view of the ground we occupied thirty years ago; but in seeing it we look *down* and not up.—*Missouri Dental Journal.*

---

